# Ethical considerations in developing an evidence base for pre-exposure prophylaxis in pregnant women

**DOI:** 10.1186/s12978-017-0425-z

**Published:** 2017-12-14

**Authors:** Kristen A. Sullivan, Anne D. Lyerly

**Affiliations:** University of North Carolina Center for Bioethics, University of North Carolina at Chapel Hill, Chapel Hill, North Carolina USA

**Keywords:** Clinical trials, Ethics, HIV, Maternal health, Pre-exposure prophylaxis, Pregnancy, Research

## Abstract

Though many women in need of access to HIV preventive regimes are pregnant, there is a dearth of data to guide these care decisions. While oral pre-exposure prophylaxis (PrEP) has been shown to prevent HIV infection in numerous high-risk populations, pregnant women have been excluded from all major prospective trials. We propose for ethical examination a theoretical trial—a prospective, observational study of PrEP for pregnant women at risk for HIV in sub-Saharan Africa—highlighting an ethical tradeoff that characterizes issues faced for advancing research in pregnancy. On the one hand, an “opportunistic” study design has certain ethical advantages: as formally construed, the research activity usually begins *after* decisions to use PrEP during pregnancy are made in the clinical setting. This minimizes research risks and avoids ethical problems that a randomized controlled trial (RCT) comparing PrEP to placebo would entail, particularly withholding care proven beneficial in other populations. On the other hand, observational studies yield less precise information than RCTs. This raises a broader question about the pace of research with pregnant women, as it typically takes many years after a drug’s approval for use in the general population to determine safety of the medication in pregnancy. Such delays can have the effect of making it impossible to ethically conduct an RCT with pregnant women, reducing the likelihood that the research community is able to obtain robust, pregnancy-specific evidence. While an observational cohort is potentially the most ethically and scientifically justified research design to study PrEP in pregnancy, earlier involvement of pregnant women in studies of newer preventives may lead to evidence that is more timely and robust.

## Case background

Approximately 17.8 million women are living with HIV worldwide [1], and millions more are at risk of infection. Given pregnancy rates in general, and that unprotected intercourse is a leading HIV risk factor for women, many women in need of HIV preventive regimes are pregnant. Additionally, incident infection with HIV during pregnancy is associated with high rates of maternal-to-child transmission [2]. Although research on prevention of maternal-to-child transmission (PMTCT) of HIV has contributed to an evidence base for antiretroviral use in pregnancy, there is a dearth of data on how to best prevent acquisition of HIV in pregnant women.

Pre-exposure prophylaxis (PrEP) has been shown to prevent HIV infection in numerous high-risk populations, yet little is known about its use in pregnant populations. Pregnancy has been an exclusion criteria from all major trials of PrEP in Africa e.g., [3, 4], and women who become pregnant while participating in such trials are required to discontinue medication. The result has been conflicting guidance on whether and when pregnant women at risk for HIV should use PrEP for prevention [5, 6]. Given the physiological changes that occur during pregnancy, research is critically needed to establish appropriate guidelines for safe and effective use of PrEP during pregnancy.

Future study, however, is uncertain due to debate about when and under what circumstances pregnant women should be involved in such research. The medications used in PrEP, including tenofovir (TDF) and tenofovir-emtricitabine (TDF-FTC) have been studied for PMTCT among pregnant women living with HIV and hepatitis B, and among women with incident pregnancy during PrEP trials; this limited data suggests these medications are generally safe in pregnancy [7–9], though further research is indicated on some outcomes [7]. On the one hand, such data are reassuring and we are now, presumably, better placed to gather robust, pregnancy-specific data. Yet the interim establishment of efficacy in non-pregnant individuals ushers in new ethical complexities, namely the challenges of gathering robust data in pregnancy after positive trials in non-pregnant populations.

We propose for consideration a prospective study of oral PrEP for pregnant women at risk for HIV in sub-Saharan Africa. While the case we propose is theoretical, a study is in fact currently under development by the International Maternal Pediatric Adolescent AIDS Clinical Trials (IMPAACT) Network, IMPAACT 2009 [10]. IMPAACT 2009 is an observational cohort study comparing pregnancy outcomes among women at risk for HIV who are taking oral PrEP to women who decline PrEP during the antenatal period. If implemented, this would be the first large prospective study of PrEP in pregnant women.

## Ethical discussion

Advancing the study of PrEP in pregnancy raises several ethical considerations in the current context, particularly ethical tradeoffs related to the choice of study design. An observational design has certain ethical advantages. First, the approach utilized is often “opportunistic”: as formally construed, research activity begins *after* decisions about use or non-use of PrEP are made in the clinical setting. Understood this way, the risks attributed to the research itself are minimal, limited to monitoring and follow-up; thus, the ethical and regulatory complexities of risk/benefit tradeoffs that often characterize interventional studies can be avoided. Second, an observational study avoids the ethical challenges that a randomized controlled study (RCT) comparing PrEP to placebo would entail, particularly withholding care proven beneficial in other populations. Indeed, a placebo-controlled RCT may now be difficult or impossible to justify given the robust evidence for PrEP in non-pregnant populations.

Yet, there are also disadvantages to such a design. Observational studies may not yield as precise data—particularly about rare events—as a randomized trial would. Thus, while an observational cohort can be an important workaround, it raises a broader question about the pace of research with pregnant women and the costs of delaying or avoiding prospective research, however ethically complex. Observational research in pregnancy most often occurs after licensure; estimates suggest that the determination of a medication’s safety during pregnancy takes an average of 27 years following the drug’s approval [11]. Delays in conducting research with pregnant women—problematic in their own right—can have the further effect of making it impossible to ethically conduct an RCT with pregnant women, reducing the likelihood there will ever be robust, pregnancy-specific evidence. To the extent delays can enroot gaps in the evidence base, they indicate an ethical cost to caution.

As we move forward in prevention efforts (and with newer technologies, such as the vaginal ring), it is important to consider not just *how* clinical trials with pregnant women could be conducted, but *when* in the research and development cycle pregnant women should be included. Clearly, delaying enrollment of pregnant women in prevention studies allows accumulation of pregnancy safety data through inadvertent exposures, and also permits the research community to avoid the ethical challenges of intentional exposure of pregnant women to interventions whose risks and benefits are unclear. However, these delays can also raise new ethical barriers to the conduct of studies that would provide robust, pregnancy-specific data. Given the costs of delay, timely inclusion of pregnant women must be addressed in developing an HIV prevention research agenda that adequately and ethically attends to the interests of pregnant women and their future offspring.

## Conclusions

There is an urgent need for HIV preventives during pregnancy; a prospective clinical trial of PrEP in pregnant women is an important step in meeting this need. While an observational cohort is potentially the most ethically and scientifically justified research design to study PrEP, earlier involvement of pregnant women in studies of newer preventives going forward may lead to evidence that is more timely and more robust. Addressing the ethical complexities of intervention research is therefore a pressing priority.

## Abbreviations

IMPAACT: International Maternal Pediatric Adolescent AIDS Clinical Trials
PMTCT: Prevention of mother-to-child transmission
PrEP: Pre-exposure prophylaxis
RCT: Randomized controlled trial
TDF: Tenofovir
TDF-FTC: Tenofovir-emtricitabine
